# The Effect of Mentoring on Career Satisfaction of Registered Nurses and Intent to Stay in the Nursing Profession

**DOI:** 10.1155/2012/168278

**Published:** 2012-05-07

**Authors:** Bette Mariani

**Affiliations:** College of Nursing, Villanova University, Driscoll Hall, Room 352, 800 Lancaster Avenue, Villanova, PA 19085, USA

## Abstract

Mentoring is important in the career development of novice and experienced nurses. With the anticipated shortage in nursing, it is important to explore factors such as mentoring that may contribute to career satisfaction and intent to stay in the profession. This study explored the effects of mentoring on career satisfaction and intent to stay in nursing, and the relationship between career satisfaction and intent to stay in nursing. It was conducted through a mailed survey of RNs 55 years or younger currently in practice, education, administration, or research. Career satisfaction was measured through the use of the newly developed Mariani Nursing Career Satisfaction Scale. Findings revealed no statistically significant effect of mentoring on career satisfaction and intent to stay in nursing. There was a statistically significant relationship between career satisfaction and intent to stay in nursing. The majority of nurses reported participating in a mentoring relationship. Although the findings related to mentoring, career satisfaction, and intent to stay were not statistically significant, there was a prevalence of mentoring in nursing, thus suggesting the need for future research to identify outcomes of mentoring. In addition, the study contributed a newly developed instrument to measure the concept of career satisfaction in nursing.

## 1. Introduction

Mentoring is important in the career development of both novice and experienced nurses in the areas of clinical practice, nursing education, administration, and research, as it supports the novice's need to feel satisfaction and success as a professional nurse and offers the experienced nurse an opportunity to contribute to the profession. This study explored the effect of mentoring on career satisfaction and intent to stay in the nursing profession, two critical elements in the retention of nurses in the profession.

Despite an encouraging recent 5.7% increase in enrollments in baccalaureate nursing programs (American Association for Colleges of Nursing (AACN)) [1], it is anticipated that the nursing shortage will continue to be a major issue in nursing in the United States for years to come. According to the AACN [1, 2], there are several factors influencing the nursing shortage, including insufficient numbers of nursing faculty, an aging nursing workforce, increasing healthcare needs of an elderly patient population, and nursing job burnout and dissatisfaction that are driving nurses away from the profession. Recent economic challenges have temporarily affected the nursing shortage and the need for nurses in some regions of the United States; however, with the combination of older nurses retiring from practice, academia, and administration, and dissatisfied nurses leaving nursing, the profession of nursing must identify strategies to increase recruitment and retention to address the nursing shortage, especially in practice and academia. Mentoring may be one such strategy. Although the shortage in any one of the areas may be viewed in isolation, there is an interdependent aspect to the shortage. A shortage in the area of clinical practice affects academia, administration, and research, and a shortage in academia, in turn, affects the clinical practice arena; therefore, these four areas of the profession were studied to determine if mentoring contributed to a greater sense of career satisfaction and intent to stay in the profession.

Several factors contribute to the shortage of nurses in the profession. National data indicate that the average age of nurses nationwide continues to increase. In 2008, the average age of nurses nationwide was 48 (HRSA) [3], and it was projected that in 2012, nurses in their 50 s will account for the largest age group of nursing workforce at about 25% of the total RN population [4]. Decreased staff satisfaction accounted for 52% of workforce shortage [5], and insufficient numbers of faculty and other factors contributed to more than 67,000 qualified applicants being turned away from baccalaureate and graduate nursing programs [1]. This along with a projected increase in demand for nursing positions calls for the profession to explore strategies to recruit and retain nurses.

The concept of mentoring is not new to nursing, as Florence Nightingale was known to have mentored many nurses in her day [6]. Stewart and Krueger [7], Yoder [8, 9], Vance and Olsen [10], Walker [11], Billings [12], Fox [13], the National League for Nursing [14], and others [15–17] have contributed to the published nursing literature on mentoring; however, further nursing research is needed related to outcomes and effectiveness of mentoring, such as career satisfaction and intent to stay in the profession.

Mentoring is a reciprocal, long-term relationship with an emotional commitment that exists between a novice (protégé) and experienced (mentor) nurse; mentoring implies a knowledge or competence gradient, in which the teaching-learning process contributes to a sharing of advice or expertise, role development, and formal and informal support to influence the career of the protégé [7, 8, 18–23]. Mentoring provides protégés and mentors with opportunities for professional growth and career satisfaction. Lack of such satisfaction with a career in nursing may contribute to nurses leaving the profession.

With the predicted shortage and anticipated need for nurses in healthcare and academia in the future, it is more important than ever to explore career satisfaction, not just job satisfaction, for nurses. Career satisfaction is defined as the contentment that a nurse feels as a nursing professional in terms of intrinsic and extrinsic rewards [24]. Career satisfaction concerns a nurse's feeling about the career choice of nursing [25]. Job satisfaction is related to the satisfaction a nurse has with a current position in nursing; career satisfaction encompasses more than just the nurses' job. It refers more broadly to satisfaction with a career in nursing and may be a critical element in retaining nurses in the profession. Nurses who have a sense of career satisfaction and feel more fulfilled may contribute to the growth of the profession. Career satisfaction was measured by total scores on the newly developed Mariani Nursing Career Satisfaction Scale (MNCSS). Mentoring may be a strategy that can contribute to career satisfaction for both the mentor and protégé.

## 2. Materials and Methods

### 2.1. Purpose of the Study

This study explored the influence of participation in a mentoring relationship on career satisfaction and on intent to stay in nursing, and the relationship between career satisfaction and intent to stay in nursing. Intent to stay in the nursing profession was defined as the nurse's plan in projected number of years to remain active in the nursing profession in practice, education, administration, or research. The following hypotheses were tested in this research study:

1. registered nurses who participated in a mentoring relationship will have greater career satisfaction than those who did not participate in a mentoring relationship;
2. registered nurses who participated in a mentoring relationship will report the intent to stay in the nursing profession longer than registered nurses who did not participate in a mentoring relationship;
3. career satisfaction will be positively related to intent to stay in the nursing profession.

For purposes of this study, the mentor was a nurse who was considered experienced, competent, or expert in the novice to expert continuum and more experienced than the novice nurse in terms of knowledge, skill, and competence. The protégé was the novice nurse in the mentoring relationship, in any area of the profession who was new to the area of practice, education, administration, or research, and lacked knowledge, experience, or competence in that setting. The mentor and protégé were self-identified on the demographic questionnaire.

### 2.2. Theoretical Framework

The theoretical works of Patricia Benner [18, 19] on novice to expert practice and Hildegard Peplau's Theory of Interpersonal Relations [20–22] were blended to provide a framework for this study. Mentoring is frequently described as an interpersonal relationship between novice and expert nurses. Benner's model, which was introduced to the nursing community in the 1980s through her acclaimed work, entitled* Novice to expert: Excellence in clinical practice *[18, 19], supports this novice to expert relationship that occurs in a mentoring relationship, and Peplau's [20–22] Theory of Interpersonal Relations supports the interpersonal phase of the mentoring relationship. Aspects of both of these theories provided support for this study on mentoring, career satisfaction, and intent to stay in nursing.

### 2.3. Review of the Literature

Mentoring has been discussed in the nursing literature as early as the days of Florence Nightingale, but more recently since 1974 with the initial work of Kramer [26]. The literature is replete with theoretical and anecdotal articles related to mentoring, although the published literature revealed limited research on mentoring related to the outcomes of career satisfaction and intent to stay in the nursing profession. Kramer, in her landmark work on reality shock, suggested that a strong mentoring process could be effective in helping new nurses move through the three phases of reality shock. Vance [23] has contributed greatly to the literature on mentoring since she began her research in 1977 on mentoring in nursing, where a sample of 71 “nurse-influentials” reported that mentoring played a key role in their success, professional satisfaction, and leadership in nursing. Vance developed a paradigm illustrating the types of mentoring that were significant to the nurse-influentials through each developmental stage of life and career, and she described the various characteristics of mentoring through the stages, including, support, guidance, teaching, counseling, advising, career support, friendship, caring, confidant, and satisfaction.

Vance and Olsen [10] and Vance [27] defined a mentoring relationship as being developmental, empowering, and nurturing, requiring commitment and self-confidence extending over a period of time, where mutual sharing, learning, and growth occur in an atmosphere of respect and collegiality. Vance [27, 28] stated the importance of strengthening mentoring in the nursing profession so that crucial aspects of the profession are retained, especially in today's healthcare environment.

Boyle and James [29] found that nurses who reported that mentors had strongly influenced their careers were significantly affected during the early years of their careers. Nurse managers (*N* = 100) were surveyed on their perceptions of mentoring experiences, expectations of mentoring relationships, organizational environment, career satisfaction, and career influence; 79% reported having a mentor at some time throughout their career, with a strong positive correlation (*r* = .78, *P* < .001) in response to a question regarding the extent to which a mentor influenced their career. The nurses who reported that mentors had a strong influence on their career believed that the most significant contributions of a mentor were offering feedback on performance, sharing expertise with the protégé, serving as a role model, and demonstrating a belief in the protégé. The findings revealed that one of the most crucial times for mentoring was early in a nurse's career and that organizational support was necessary.

Yoder's [8] concept analysis of mentoring described aspects of mentoring, such as coaching, challenging assignments, protection, sponsorship, exposure, visibility, competence, effectiveness of role acquisition, role modeling, and friendship within two dimensions: that of career functions and psychosocial functions. In her concept analysis of mentoring, Yoder identified two critical antecedents (the mentor and protégé), three critical attributes of mentoring (a structural role, an organizational phenomenon, and a career development relationship), and three outcomes (personal satisfaction, self-confidence, and empowerment) [8].

Stewart and Krueger's [7] concept analysis of mentoring was built upon Yoder's work in 1990, identifying six critical attributes of the concept: a teaching-learning process, a reciprocal role, a career development relationship, a knowledge or competence differential between participants, a duration of several years, and a resonating phenomenon; they also identified the following consequences of mentoring: career progression, development of new investigators, empowerment, expanding professional knowledge and practice base, generativity, increasing numbers of minority nurses in master's and doctoral programs, institutional stability, and professional socialization [7]. Dunham-Taylor et al. [30] cited mentoring of new faculty as the best method to influence retention and successful development of nursing faculty. Despite the literature that is available on mentoring, gaps exist in empirical evidence on outcomes of mentoring, such as career satisfaction and intent to stay in the profession.

### 2.4. Description of the Study

This study used a combination of a descriptive comparative and correlational design. The phenomenon of interest in this study was mentoring, the independent variable was participating versus not participating in a mentoring relationship as a protégé or mentor, and the dependent variables were career satisfaction and intent to stay in nursing.

The study was conducted through the use of a written survey. Professional nurse databases that were available for scholarly use or purchase from the State Boards of Nursing were used to access names and addresses of RNs. The target population included RNs aged 55 or below, who were currently employed in the practice, education, administration, or research setting. The age of 55 or less was selected because nurses who are over 55 may be considering retirement which could have skewed the results. Since the aging workforce in nursing is partly responsible for the predicted shortage, the aim of this study was to explore the intent of younger nurses to stay in nursing.

In addition, the intent was to study nurses who were currently employed in nursing practice, education, administration, or research; therefore, the letter of explanation describing the study included current employment as a criterion for inclusion in the study. The demographic questionnaire also addressed the current employment setting of the study participants and their educational level.

A survey was used in this study in an effort to reach a large number of nurses throughout the country; the disadvantage was that the rate of return for a survey or questionnaire is often low [31]. Colored paper and 5(3/4) × 8(3/4) inch colored envelopes were used in an effort to improve the visibility of the surveys in the mail and the response rate. In addition, distribution of the surveys to a larger population increased the likelihood of an adequate sample size.

### 2.5. Sample Selection and Sample Size

To obtain a national random sample throughout the United States, the country was divided into the nine geographic regions (see Table 1) that were used for the national sample survey of registered nurses [3]. One state was selected from each region by clustered random sampling, and the state board of nursing was contacted to obtain the mailing lists. After the lists were obtained, random sampling was used to mail the surveys to 60 RNs from each state in the first mailing, and a second mailing was sent to 15 different RNs from each state two weeks later in an effort to increase the likelihood of more surveys being returned. In addition, after the planned sampling procedure failed to yield a sufficient subsample of nurses who did not participate in a mentoring relationship, a convenience sample from a small private university in the Middle Atlantic region was used in an attempt to obtain an adequate sample size. Although the purpose of the convenience sample was to try to increase the number of responses for the group who did not participate in a mentoring relationship, this additional sample only yielded more subjects who had participated in a mentoring relationship.

The required sample size of 102 subjects was determined based on a power analysis for both the *t*-test and the Pearson Correlation, using a power of  .80 and a moderate effect size for use of  *t*-tests and Pearson's correlation. For this study, the total sample was 173, but the group size for those RNs who did not participate in a mentoring relationship was only 37; therefore, the actual power in this study was  .70 [32].

### 2.6. Instrumentation

 A demographic questionnaire and a newly developed, pilot-tested research instrument, the Mariani Nursing Career Satisfaction Scale (MNCSS), was used for data collection. The MNCSS developed by this researcher measured career satisfaction. The entire survey took approximately 20 minutes for the subjects to complete. The demographic questionnaire addressed the nurse's age, gender, highest educational level, current employment status, years in practice, education, administration, or research, participation in a mentoring relationship, intent to stay in nursing (in years), whether the participant would choose nursing again and would recommend nursing as a career.

 Although, there were several valid and reliable instruments, such as the Stamps and Piedmonte Index of Work Satisfaction [33], to measure job satisfaction, a review of the literature revealed no valid and reliable instrument to specifically measure nurses' career satisfaction. Therefore, a new instrument that more accurately represented the intent of this study, career satisfaction in the nursing profession, was developed.

The MNCSS is a semantic differential scale with 16-bipolar adjective pairs on which the participants rated their feelings about their nursing career that best represented their attitude toward the adjective. For the MNCSS, each adjective pair was scored on a seven-point scale using the lower number for the less positively worded adjective and the higher number for the most positively worded adjective. An example of some of the 16 adjective pairs is satisfied-dissatisfied, fulfilled-discouraged, stimulated-bored, content-discontent, rewarded-frustrated, meaningful-not meaningful, and successful-unsuccessful. The sum of the point values of responses to all 16 items yielded a total MNCSS score. The total scores could range from 16 to 112 with a midpoint of 64. The overall content validity index (CVI) of the MNCSS was  .84 for the 16-bipolar adjectives that were included as part of the instrument. Cronbach's alpha internal consistency reliability of the instrument was  .82 for the pilot study, and  .94 for the full study.

 Prior to data collection, IRB approval was obtained. The participants received the demographic questionnaire and the MNCSS with a letter of explanation including the intent of the study and the definitions of key terms. Consent was implied by the participant's return of the completed questionnaires. A stamped, return envelope was included to mail the questionnaire back to the researcher. The participants were given information as to how they could access results of the study, if desired.

Of the total 722 surveys that were mailed and distributed, 196 surveys were returned, and 173 of the surveys were usable for the research study. Of the 23 surveys that were not used 6 were not included because the respondents exceeded the age of 55 years of age, 10 were not currently employed in practice, education, administration, or research, 6 were returned after the final due date, and 1 did not complete the career satisfaction scale.

## 3. Results and Discussion

### 3.1. Results

 The statistical analyses were determined by the research hypotheses. For the MNCSS, a subject with more than two missing data bits was removed from the total sample. For two subjects who were missing a response to one item on the MNCSS, the missing data were replaced with the group mean [34].

 Frequency and percentage statistics were computed for the variables of gender, education level, and area of practice and are presented in Table 1. The descriptive statistics, including mean, median, standard deviation, and range, were computed for the variables of age, years of practice, and years in nursing and are presented in Table 2. The average age of participants was 41.25 years, the youngest subject was 22 years old and the oldest was 55 years old. The mean number of years of work experience was 14.07 years, with the mean numbers of years in the clinical setting of 11.28 years. Further details of these descriptive statistics are presented in Table 2.

Of the total sample (*N* = 173), 78.6% reported participating in a mentoring relationship, 41% of these mentoring relationships were informal; 21.4% of the RNs had not participated in a mentoring relationship. From the total sample, 44.5% had been in the role of both mentor and protégé at some point in their career, not just a mentor or a protégé. The majority of RNs (*n* = 145, 83.8%) responded that they would choose nursing as a career again, and 152 (87.9%) of these RNs responded that they would recommend nursing as a career to others (Table 1).

Hypotheses 1 and 2 were tested using independent *t*-tests to determine the differences in career satisfaction and in intent to stay in nursing between the two groups, those who participated in a mentoring relationship and those who did not. In addition, Levene's analysis was computed to test the assumption of homogeneity of variance [31, 34, 35]. Hypothesis 3 was tested using Pearson's correlation.

For the total sample of 173 RNs, the overall mean for the MNCSS was 89.05 (SD = 14.33) with a range of 46 to 112 (Table 3). For hypothesis 1, there was no statistically significant difference on the MNCSS between nurses who participated in a mentoring relationship (*n* = 136; *M* = 89.79; SD = 13.94) and nurses who did not participate in a mentoring relationship (*n* = 37; *M* = 86.35; SD = 15.56). The mean number of years for intent to stay (*n* = 167) was 18.51 years (SD = 8.38). For hypothesis 2, there was no statistically significant difference in the number of years nurses intended to stay for those who did or did not participate in a mentoring relationship. Additional details of the statistics for career satisfaction and intent to stay are presented in Tables 4 and 5. For hypothesis 3, the correlation between career satisfaction and intent to stay in the profession was positive (*r* = .15) and statistically significant (*P* < .03, 1-tailed), however it was a weak correlation.

 Additional statistical analyses were computed using a one-way ANOVA to compare types of mentoring (formal, informal, both, and none) on career satisfaction and intent to stay, and a one-way ANOVA compared the types of the roles in a mentoring relationship (mentor, protégé, both, or none) on career satisfaction and intent to stay; however, there was no statistical significance found in any of the additional analyses.

### 3.2. Discussion

The differences in RNs' career satisfaction and intent to stay in the profession were not statistically significant for those who participated or did not participate in a mentoring relationship, regardless of whether it was a formal or informal relationship or if they were a mentor or protégé. Although 78.6% of the nurses surveyed indicated some type of participation in a mentoring relationship, the largest percentage of mentoring occurred informally. This suggests that nurses may create informal mentoring relationships whether or not there is an opportunity for a formal mentoring relationship. These findings are consistent with what has been reported for several decades in the mentoring literature in nursing, which points to the prevalence of mentoring relationships in nursing [8, 10, 12, 14–17, 23, 27, 28, 30, 36–40]. The nursing literature indicated that mentoring occurred in various settings in the profession, such as clinical practice, education, administration, and research, although there was little literature to support career satisfaction as an outcome of mentoring relationships. In this study, the phenomenon of mentoring was identified by the majority of the participants which may suggest that nurses place value in mentoring; therefore, researchers need to identify better measures of the outcomes of mentoring relationships.

Overall, the RNs in the sample reported generally moderate to high career satisfaction, regardless of whether or not they participated in a mentoring relationship. Although participation in a mentoring relationship did not have a statistically significant influence on career satisfaction, the majority of nurses surveyed participated in these relationships, which suggests that the value of mentoring may lie not in career satisfaction, but in other outcomes that were not measured in this study.

Although, there was limited literature on intent to stay in the profession, Krozek [41] indicated that 35% to 60% of nurses nationwide were leaving nursing within the first year of their career. Since the average age in the current study was 41.25 and this population intended to stay for almost another 20 years, it is likely that there are reasons other than mentoring that contributed to nurses staying in the profession longer, such as job security, the altruistic value of nursing, the caring nature of nursing, or the lack of other career options. Although participation in a mentoring relationship was not a significant factor in intent to stay, with the current shortage of nursing and the aging of the nursing workforce, it is encouraging to see that RNs intended to stay for almost 20 more years. However, since this study did not capture the younger population of nurses as was hoped, it is possible that the results may have been different if the participants were younger.

The findings of this research did not support the research hypotheses 1 and 2. It is possible that nurses who were not satisfied with their career or did not participate in a mentoring relationship or planned to leave their career in nursing may have elected not to participate in the study. In addition, the mean age plus the overall high satisfaction scores of the sample may have influenced the overall results of the study, and the MNCSS may not have been sensitive enough to detect a difference between the groups. The sample of nurses in this study, although consistent with the age reported in the literature at the time of the study, represented nurses who may be more than halfway through their career; therefore, the results of this study may not be representative of the younger population of nurses. For hypothesis 3, the correlation of career satisfaction to intent to stay in nursing was weak (*r* = .15).

A large number of participants in this study indicated that they had participated in an informal mentoring relationship; although the definitions were provided to the participants in the letter of explanation, the possibility exists that the participating nurses may not have differentiated role modeling, coaching, and precepting as concepts different from informal mentoring.

There were several limitations noted in this study. A modified Total Dillman Method (TDM) was used to distribute the surveys; however, there was only a 27% overall return rate. While this was not extremely low, the rate of return did not reach the projected 60% that Dillman [42–44] suggested could be achievable by multiple contacts with the intended study population. Due to the cost of multiple mailings, it was not possible to send out four mailings, as suggested by the TDM. In addition, Dillman recommended a short letter of explanation. This was not possible for this study due to the nature of the definitions that were necessary to include for the intended study population.

Another limitation may have been attributable to the overall population that received the survey. The database lists for RN names and addresses were not as accessible as expected, and since the names were randomly selected from the lists, it was not possible to know in advance if those who received the survey actually met the inclusion criteria for the study of being currently employed in clinical practice, education, administration, or research despite having an active RN license and meeting the age criterion. In addition, the random search for names nationwide was a time intensive and financially expensive process.

Another limitation pertained to the unevenness of the survey response rate from those who had participated in a mentoring relationship (*n* = 136) and those who had not participated in a mentoring relationship (*n* = 37). It was difficult to achieve an adequate sample size for RNs who had not participated in a mentoring relationship. From the 722 surveys that were mailed, there was only a 5.12% rate of return for the group that had not participated in a mentoring relationship. This may have been attributable to the following reasons: RNs who were not in a mentoring relationship did not participate because they thought the survey pertained only to RNs who had been in a mentoring relationship; RNs who had not participated in a mentoring relationship are no longer in nursing; or there is more mentoring occurring in the profession than is typically thought. Although a mentoring relationship at any point in a nurse's career may have significance, the question on the survey pertaining to participation in a mentoring relationship did not ask when the mentoring relationship occurred; therefore, the mentoring relationship may have occurred recently or 20 or more years ago.

In addition, the survey question that asked the study participant about participation in a mentoring relationship was a self-assessment of their participation and role in a mentoring relationship. Although the distinctive definitions of a preceptor, a mentor, and a protégé were included in the letter of explanation, participants who responded positively to participating in an informal mentoring relationship may have been referring to a precepting relationship or role modeling, not necessarily a mentoring relationship in the truest form of the definition. And lastly, RNs who were dissatisfied with nursing as a career may not have been interested in participating in a research survey.

There are implications for nursing related to this study's findings. With the predicted shortage of nurses, the implications of this study and the recommendations for future studies may have an effect on nursing practice, education, administration, research, and science. Although the results of this study were not statistically significant related to the outcomes of career satisfaction and intent to stay, 136 of the 173 participants indicated that they had participated in a mentoring relationship either formally or informally as a mentor or protégé at some point in their career. Nurses should continue to encourage mentoring relationships within the profession to foster some of the other positive outcomes of mentoring such as personal satisfaction, self-confidence and empowerment [8], and professional development [45, 46]. Mentoring may help bridge the gap between novice and expert practice and help new nurses feel satisfied with nursing as a profession, increase retention and, hopefully, decrease the impact of the nursing shortage. Kalagher [47] wrote that nurse leaders were in a position to positively influence the careers of novice and expert nurses. Mentoring and career satisfaction could contribute to retention of registered nurses in the profession, while providing an opportunity to groom future nurse leaders. Mentoring programs may be costly, but orientation and turnover in the profession are also costly; therefore, retention of nurses in the profession through mentoring programs may be a cost reducing measure for administrators. Leaders are in a position to influence the development of more formal mentoring programs by validating the effectiveness and financial impact of such programs.

 Although, this study did not yield statistically significant results supporting the outcomes of career satisfaction and intent to stay in the profession with a mentoring relationship, the presence of the phenomenon of mentoring was very apparent. Through this study, future research initiatives can be identified to explore the outcomes of mentoring. In addition, this study contributed to nursing science and research through the development of a new valid and reliable nursing research instrument, the MNCSS, to measure the concept of career satisfaction in nursing.

This study demonstrates that mentoring is a prevalent phenomenon in nursing. Mentoring is a concept that is well-supported in the literature; research that continues to explore mentoring relationships in nursing can help demonstrate the positive outcomes of mentoring in nursing.

## 4. Conclusions

This study explored whether participation in a mentoring relationship had a positive effect on career satisfaction and intent to stay in the nursing profession. Based on recent statistics in the literature, despite increasing nursing school enrollment, it is predicted that the nursing shortage will continue. This study did not demonstrate a statistically significant difference in career satisfaction and intent to stay between nurses who participated in a mentoring relationship and nurses who did not participate in a mentoring relationship. However, there was a statistically significant positive relationship between career satisfaction and intent to stay in the nursing profession.

Despite the findings, this study contributes to the body of knowledge for the nursing profession related to mentoring relationships, nurses' career satisfaction, and intent to stay in the nursing profession. Nurses responded favorably about their careers in nursing and the importance of mentoring relationships which is promising for the profession of nursing. Further theory development and research are needed to add to the body of knowledge about the outcomes of mentoring related to career satisfaction and intent to stay in nursing.

## Figures and Tables

**Table 1:** Descriptive statistics of categorical demographic data (*N* = 173).

Demographics	Frequency^a^	Percent
Gender (*n* = 173)		
Female	150	86.70
Male	23	13.30

Region (*n* = 173)		
New England	16	9.20
Middle Atlantic	48	27.70
South Atlantic	16	9.20
East South Central	14	8.10
West South Central	12	6.90
East North Central	15	8.70
West North Central	16	9.20
Mountain	8	4.60
Pacific	28	16.20

Highest level of nursing education (*n* = 173)		
Diploma	11	6.40
Associate's degree	61	35.30
Bachelor's degree	70	40.50
Master's degree	31	17.90

Currently enrolled in advanced degree (*n* = 173)		
Yes	48	27.70
No	125	72.30

Current area of practice (*n* = 171)^b^		
Clinical setting (*n* = 154)		
Full-time	114	65.90
Part-time	40	23.10
Education (*n* = 31)		
Full-time	16	9.20
Part-time	15	8.70
Administration (*n* = 26)		
Full-time	19	11.00
Part-time	7	4.00
Research (*n* = 2)		
Full-time	1	0.60
Part-time	1	0.60

Mentoring relationship (*n* = 173)		
Total yes	136	78.60
Formal	25	14.50
Informal	71	41.00
Both	40	23.10
None	37	21.40

Role in mentoring relationship (*n* = 170)		
Mentor	32	18.50
Protégé	24	13.90
Both	77	44.50
None	37	21.40

Choose nursing again (*n* = 172)		
Yes	145	83.80
No	27	15.60

Recommend nursing (*n* = 172)		
Yes	152	87.90
No	20	11.60

^a^Totals do not include missing data.

^b^ Two of the subjects did not indicate an area of current practice.

**Table 2:** Descriptive statistics of continuous demographic data.

Demographics	*n*	Mean	SD	*Md*	Range
Age	173	41.25	9.10	43.00	22 to 55
Years of RN work experience	173	14.07	9.57	14.07	0 to 34
Area and years of current practice					
Clinical practice	146	11.28	8.29	11.28	0 to 34
Education	30	5.80	5.37	5.80	1 to 27
Administration	25	6.56	5.18	6.56	1 to 20
Research	2	9.00	1.41	9.00	8 to 10

**Table 3:** Mean scores of career satisfaction and intent to stay in nursing.

Variable	*n*	Mean	SD	Median	Range
MNCSS total	173	89.05	14.33	92.00	46 to 112
Mentoring	136	89.79	13.94	93.00	46 to 111
No mentoring	37	86.35	15.56	86.00	53 to 112

Intent to stay total	167	18.51	8.38	20.00	0 to 40
Mentoring	132	18.44	7.93	20.00	2 to 40
No mentoring	35	18.80	10.02	20.00	0 to 35

**Table 4:** Mean scores of career satisfaction and *t*-test comparing mentoring relationship and career satisfaction.

MNCSS score	*n*	Mean	SD	*t*	*df*	*P*
Mentoring Relationship	136	89.79	13.94	1.30	171	.098
No mentoring Relationship	37	86.35	15.56			

**Table 5:** Mean scores of intent to stay and *t*-test comparing mentoring relationship with intent to stay.

Intent to stay	*n*	Mean	SD	*t*	*df*	*P*
Mentoring Relationship	132	18.44	7.93	−.20	45.91	.42
No mentoring Relationship	35	18.80	10.02			
